# Highly variable soil dissipation of metaldehyde can explain its environmental persistence and mobility

**DOI:** 10.1016/j.chemosphere.2021.131165

**Published:** 2021-11

**Authors:** Nathan Keighley, Carmel Ramwell, Chris Sinclair, David Werner

**Affiliations:** a Fera Science Ltd., York Biotech Campus, Sand Hutton, York, YO41 1LZ, UK; b School of Engineering, Newcastle University, NE1 7RU, Newcastle upon Tyne, UK

**Keywords:** Molluscicide, Metaldehyde, Biodegradation, Pollution, Surface water

## Abstract

There are increasing concerns about the hazard posed to drinking water resources by persistent, mobile, and toxic (PMT) substances in the environment. For example, the extensive use of metaldehyde-based molluscicide to control slug populations in agricultural fields has frequently led to pollution of surface waters and contamination of drinking water at levels exceeding the statutory limit. Regulatory environmental fate assessments and studies in the literature did not predict that metaldehyde would be persistent in the environment, contrary to observations from monitoring schemes. To understand the reasons for this disparity, this study conducted a suite of degradation experiments, covering different soil types and environmentally realistic conditions in Northern Europe, and generated a distribution of DT_50_ values for metaldehyde to examine whether degradation rates are underestimated by current risk assessments. The results were found to vary, showing DT_50_ values ranging from 3.0 to 4150 days, which indicated that metaldehyde had the potential to become persistent. Lack of prior metaldehyde exposure, high moisture content, low temperature, and locally high metaldehyde concentration under pellets were identified as high-risk conditions for low pesticide biodegradation in UK soils.

## Highlights

• Metaldehyde, a toxic, mobile and apparently persistent compound was investigated.
• The Arrhenius equation poorly fitted metaldehyde biodegradation rates.
• Metaldehyde was persistent in pasture soil at high soil moisture contents.
• High metaldehyde concentrations reduced potential for degradation.
• These observations rationalize metaldehyde persistence and leaching risks.

## Graphical abstract

**Figure:**
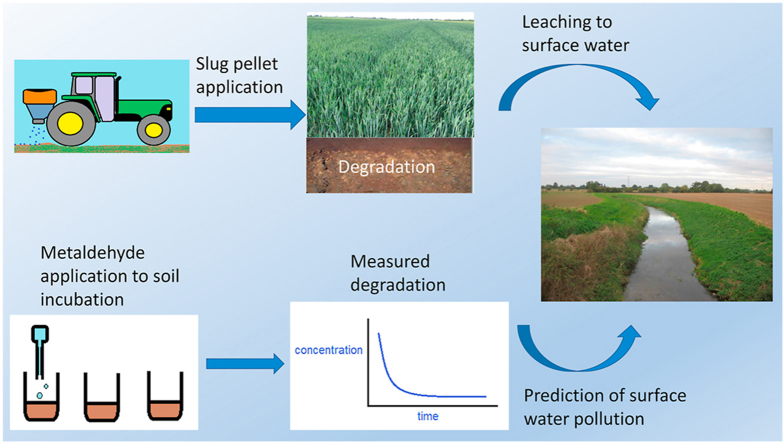

## 1 Introduction

In Europe there are increasing regulatory activities to address concerns about persistent, mobile, and toxic (PMT) substances in the environment (Rüdel et al., 2020; Hale and Arp, 2020a, Hale and Arp, 2020b). For example, widespread use of molluscicide has resulted in the active ingredient metaldehyde being frequently detected in drinking water in exceedance of the current statutory limit of 0.1 μg L^−1^ (Drinking Water Inspectorate, 2018). Current methods to remove metaldehyde from drinking water, for example by using existing granular activated carbon treatment methods, are ineffective and too expensive to be commercially viable (e.g. Salvestrini et al., 2017; Busquets et al., 2014). This has necessitated catchment-based interventions and modelling of metaldehyde concentrations in surface waters to predict peak concentrations and manage abstraction times (Asfaw et al., 2018, Castle et al., 2017, Kay and Grayson, 2014, Lu et al., 2017). These models rely on input parameters, such as pesticide degradation rates, derived from laboratory experiments. Standard degradation tests used for regulatory risk assessment, such as OECD 307, did not predict that metaldehyde would be a pollutant of concern (EFSA, 2010). A peer review of the pesticide risk assessment of the active substance metaldehyde conducted on behalf of the European Food Safety Authority (EFSA) concluded that metaldehyde exhibited high to very high mobility in soil, with K_FOC_ values ranging between 38 and 149 mL g^−1^, but the compound was assessed as exhibiting low persistence in the aerobic soil environment with DT_50_ values ranging between 2.6 and 6.7 days (EFSA, 2010). Hence the compound was not considered persistent in the soil environment. Furthermore, metaldehyde was assessed as harmful to aquatic organisms (including gastropods) based on the data available, but based on predicted environmental concentrations, the risk to aquatic organisms and groundwater resources was assessed as low (EFSA, 2010). However, the frequent detection of metaldehyde in surface and drinking water in the UK (Drinking Water Inspectorate, 2018) does not agree with these risk assessments. This study aimed to investigate the cause of this disparity.

Metaldehyde enters water sources primarily as a diffuse pollutant from agricultural fields. As a small and polar molecular with chemical formula C₈H₁₆O₄, molar mass 176.2 g/mol, an octanol-water partitioning coefficient log K_ow_ of 0.12 at 20 °C, and 0.188 g L^−1^ at 20 °C water solubility (Castle et al., 2017), metaldehyde readily dissolves in water and has a relatively low binding affinity with soil (Castle et al., 2017, Castle et al., 2018). In water and low oxygen environments it remains very stable, and therefore in reservoirs, metaldehyde concentrations do not decrease readily (Castle et al., 2018). The only significant route by which metaldehyde is effectively removed from the environment is through biodegradation in soil. Several environmental fate assessments for metaldehyde in the literature (e.g., Zhang et al. (2011); Dong et al. (2017); Calumpang et al. (1995); Bond (2018)) measured fast degradation in soil, when using the standard regulatory methods according to OECD 307, and therefore suggested that the compound should not be a persistent pollutant of concern. The main hypothesis of this investigation was that soil degradation rates for metaldehyde are overestimated by current risk assessments.

Standard guidelines for examining pesticide degradation in soil recommend an incubation temperature of 20 °C and soil moisture content of 60% maximum water holding capacity (MWHC). These conditions offer a standardised test that allows for some market alignment in the chemicals industry and a starting point for consistently carrying out assessment of risks across Europe. However, these parameters are not necessarily representative of environmental conditions in the UK when slug pellets are typically applied, i.e., in spring and autumn, when the average temperatures are 8.7 °C and 9.9 °C, respectively (Met office, 2020). This investigation tested metaldehyde degradation in three UK soil types over a range of temperatures, soil moisture contents, and metaldehyde concentrations, taking into consideration that metaldehyde is applied as pellets. In conventional degradation tests it is assumed that the pesticides are homogenously applied across a field, which is a reasonable assumption for application as a spray. But for pesticide application in pelletized form, a patchier distribution pattern will result in discrete areas of high pesticide concentration in the soil around each pellet. The aim was thus to improve understanding of metaldehyde degradation in soil for variable environmental conditions comparing standard OECD conditions to those that are more realistic in Northern Europe. Pesticide degradation at lower temperature, more representative of the UK scenario, is usually estimated in models using the Arrhenius equation (Matthies and Beulke (2017); EFSA (2007)). Uncertainty about the actual temperature dependency of metaldehyde degradation was hypothesised to be a possible source of error that might lead to an overestimation of metaldehyde degradation in the field. Also, during slug pellet application season, soils in the UK are likely to be much wetter than 60% MWHC. It was hypothesised that the reduced availability of air for aerobic mineralisation processes in highly moist soil would lead to reduced degradation of metaldehyde. The third hypothesis was that metaldehyde degradation rates might be overestimated by postulating that metaldehyde degradation would not be affected by its concentration in soil. The degradation kinetics of metaldehyde were therefore also investigated at higher concentrations, consistent with the application of the compound in pellets, giving rise to localised, discrete areas of high concentrations.

## 2 Materials and methods

### 2.1 Batch degradation experiments

A series of batch degradation experiments were conducted to identify whether varied experimental conditions, more representative of the range of field conditions in the UK and Northern Europe, would significantly affect the degradation of metaldehyde in soil. Batch degradation studies were performed with three field fresh soils collected from the same district in North Yorkshire: a clay loam (CL) soil collected from a permanent pasture field on 02/07/19, an arable clay loam collected on 08/11/19, and an arable sandy loam (SL) collected on 16/01/20. The pasture clay loam was taken from a field that had historically been used for sheep farming and was therefore free of any residual metaldehyde, based on land-use history. The arable clay loam was of the same texture class (refer to Table 1), collected from the adjacent field. It was selected to investigate how land management practices might influence biological processes occurring in the soil. The field had been used for growing wheat in the previous harvest. The sandy loam was selected from a local field to represent a contrasting soil type to elucidate whether any observed trends in metaldehyde degradation would continue in other soil types. This field also had wheat as the previous crop.

**Table 1:** Properties of the soils collected from the field for use in the batch degradation experiments.

Soil sample	MWHC (%)	pH (CaCl_2_)	OC %	Sand %	Silt %	Clay %
Pasture CL	34.4	6.3	2.7	43.0	29.0	28.0
Arable CL	32.6	6.0	3.3	29.0	34.0	37.0
Arable SL	26.4	5.5	1.8	48.0	32.0	20.0

CL = clay loam; SL = sandy loam.

The batch degradation experiments tested the effects of 1) temperature, 2) soil moisture content and 3) metaldehyde concentration on degradation rates, compared to a reference experiment conducted in accordance with OECD 307 guidelines at an incubation temperature of 20 °C and soil moisture of 60% MWHC. The experimental methods to test each of these parameters were kept the same, other than for the use of a different soil type. Metaldehyde (an appropriate aliquot of a 0.1 g L^−1^ aqueous solution) was dosed into soil (20 g or 50 g) in accordance with OECD 307 guidelines to achieve 2 mg kg^−1^ fortification, unless otherwise stated. The soil moisture content was adjusted to the required value using deionised water, based on the sample weight, and amended weekly. Moisture content fluctuations in a week were only 5% at the most, so unlikely to affect degradation rates. Metaldehyde was extracted, in accordance with the validated method, 0, 1, 3, 7, 14, 21, 28 and 60 days after treatment and subsequently quantified using LC-MS.

Using the experimental procedure described above, metaldehyde degradation was measured in soils at five temperatures. The batch soil samples were assembled, dosed and then moved to separate incubators at temperatures of 20 °C, 16 °C, 12 °C, 8 °C and 4 °C to test the effect of decreasing temperature on the degradation rate of metaldehyde. Degradation experiments at different soil moisture contents were also assembled. A degradation experiment using the pasture clay loam was set up with the soil moisture content amended to 100% MWHC, incubated at 20 °C. Further experiments to test the effect of soil moisture content on metaldehyde degradation were set up using the arable clay loam. The field fresh soil was allowed to dry to a moisture content of 40% MWHC in trays before been weighed into containers, and, in separate experiments, adjusted to moisture contents of 40%, 60%, 80% and 100% MWHC, with a further two experiments (soil moistures of 60% and 100% MWHC) incubated at 12 °C. Experiments with soil moisture contents of 40%, 60% and 100% MWHC were replicated in the sandy loam soil. Additionally, experiments were set up with varying metaldehyde application doses: 100 mg kg^−1^ dry soil (pasture and arable clay loam; arable sandy loam), compared to 2 mg kg^−1^ used in the standard test paradigm, and 50 mg kg^−1^ soil (arable sandy loam only). The fortification concentration of 100 mg kg^−1^ soil equates to the amount of metaldehyde contained within a single 3% slug pellet (0.375 mg active ingredient, e.g., AXCELA pellets) on a square centimetre of soil and 2.5 cm depth, i.e., representative of field conditions. The metaldehyde was applied as a solid powder mixed into silica sand rather than as an aqueous solution because the amount being applied would not readily dissolve in water. An argument can be made that spiking a solid formulation would not be immediately bioavailable, compared to a ready-dissolved formulation, but the objective of the experiment was to be more realistic, as metaldehyde is applied to soil as a solid pellet. A summary of the various experimental conditions is presented in Table 2. Each degradation experiment comprised 3 replicates plus an untreated control. Additionally, a degradation experiment was conducted using soil that had been sterilised in an autoclave to serve as a control.

**Table 2:** Summary of the batch degradation experiments; experimental conditions and calculated disappearance half-lives (DT_50_) using Single First Order (SFO) kinetics.

Soil type	Soil moisture (% MWHC)	Fortification level (mg kg^−1^)	Incubation temperature (⁰C)	DT_50_ (days)
Pasture clay loam	60	2	20	46.5
	60	2	16	46
	60	2	12	58.3
	60	2	8	71.7
	60	2	4	129
	100	2	20	4150
	60	100	20	116
Arable clay loam	60	2	20	3.0
	100	2	20	5.6
	80	2	20	3.6
	40	2	20	13.8
	100	2	12	9.3
	60	2	12	14.7
	60	100	20	7.4
	60	50	20	7.8
	60	2	20	>10,000^a^
Arable sandy loam	60	2	20	10.8
	100	2	20	7.7
	40	2	20	4.5
	60	100	20	13.5

a Sterile control.

### 2.2 Data manipulation

**Fig. 1:**
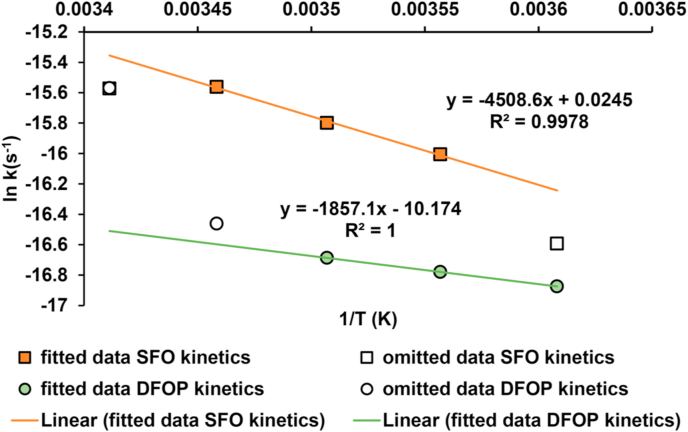
Arrhenius plots derived from the linear range of the experimental results from the batch incubation tests conducted over five temperatures from 4 to 20 °C, using Single First Order (SFO) kinetics and Double First Order Parallel (DFOP) kinetics to derive an activation energy for metaldehyde degradation in pasture clay loam soil.

The measured degradation data from individual time points were analysed using the software, Computer Assisted Kinetic Evaluation (CAKE) (Tessella, ALTRAN GROUP) to calculate degradation rates. The measured temperature-effect data were used to investigate the accuracy of the Arrhenius model which is commonly used in environmental fate models to extrapolate from temperatures used to conduct standard laboratory fate studies (20 °C and 25 °C) to field conditions. The Arrhenius equation was used to construct the plots in Fig. 1, from which the value of the activation energy was derived. This value was then used to predict the degradation rates at temperatures <20 °C and compared with the predictions using the standard activation energy that is utilised in regulatory fate models. The Arrhenius model was then extended to different soils and varied soil moisture contents to examine how predicted degradation rates might differ under different test conditions.

### 2.3 Analytical method and validation

The extraction method for metaldehyde analysis was validated against a range of standard soils (Lufa-Speyer), covering different texture classes and organic matter contents. These were selected to validate the extraction method for a range of soil properties with high recovery (70–110%). Details of the method validation are given in the accompanying MethodsX paper. Soil samples (20 g dry weight basis) were weighed into centrifuge tubes and moisture adjusted to 60% MWHC. Three replicates were used as well as an untreated control. The samples were spiked with metaldehyde (Sigma Aldrich, 99% purity) at two fortification levels (0.03 mg, as 300 μl of 0.1 mg mL^−1^ stock solution; 0.003 mg as 300 μl of 0.01 mg mL^−1^ stock solution), thoroughly mixed with a spatula, and extracted with 25 ml methanol on a side-to-side shaker (200 rpm for 30 min), centrifuged (5 min at 3500 rpm) and the supernatant poured off and stored in a vial (extract A). This process was repeated for a second sequential extraction (extract B). The extracts A and B were stored in separate vials in a refrigerator at 4 °C. The fortification concentrations of 1.5 mg kg soil^−1^ and 0.15 mg kg soil^−1^ were chosen as being approximately the same as the fortification concentration for the batch experiments, then a tenth of that value, respectively. Further validation samples were produced at a one-hundredth dose (0.015 mg kg^−1^), to determine the limit of quantification (LOQ) for the LC-MS instrument to detect metaldehyde in soil extracts, which was found to be 0.1 μg L^−1^. The recoveries obtained, which ranged between 100.4 and 132.2%, median 106%, are given in the accompanying MethodsX paper.

### 2.4 LC-MS analysis methodology

Samples for LC-MS were prepared by diluting 1/10 with HPLC grade water and filtered through a 0.2 μm pore nylon filter (*Whatman, Thermo Fisher Scientific*). Calibration standards were matrix matched, produced from the same metaldehyde stock solution used for spiking batch experiments, spiked with blank soil extract. The calibration range used included the following standards: 0.01 μg ml^−1^, 0.05 μg ml^−1^, 0.1 μg ml^−1^, 0.25 μg ml^−1^, 0.5 μg ml^−1^, 0.75 μg ml^−1^, 1.0 μg ml^−1^, and 2.5 μg ml^−1^.

The LC-MS method was developed from the one previously described (Thomas, 2016) and optimised for metaldehyde in soil extracts. Details of the alterations to the original paper are given in the accompanying MethodsX paper. The column was the BEH phenyl (Acquity UPLC) 1.7 μm particles 2.1 × 100 mm column, with a flow rate of 0.4 ml min^−1^, using a mobile phase comprising 1 mM ammonium acetate, prepared with ultrapure water, and methanol as the organic component. Samples were stored in a refrigerator at 4 °C prior to injection into the instrument. Mass spectrometry used 5.25 kV capillary voltage and 325 °C solvation temperature. To observe product ions, multiple reaction monitoring with a dwell time of 160 ms was used.

## 3 Results and discussion

### 3.1 Overview of the batch degradation experiments

The measured degradation rates for metaldehyde were lower for the permanent pasture clay loam soil, while in the arable soils, metaldehyde was shown to degrade quickly, in line with findings from the literature (EFSA, 2010). The batch degradation experiments demonstrated a highly variable set of degradation rates with DT_50_s ranging from 3.0 to 4150 days, compared to 2.6–6.7 days in the EFSA peer review report (EFSA, 2010). These could not always be linked to the parameter under investigation, and the greatest variability, generally, was observed between soil types as opposed to the other tested parameters. It was found that Single First Order (SFO) kinetics was the best fitting model for calculating degradation rates in each experiment. The most conspicuous result was that of the pasture clay loam under high soil moisture (100% MWHC) conditions, where the DT_50_ was two orders of magnitude greater than the respective reference experiment at 60% MWHC moisture content. This demonstrated that metaldehyde had the potential to become persistent in high moisture content soil without prior metaldehyde exposure.

### 3.2 Temperature dependency of metaldehyde degradation

The temperature-dependency of metaldehyde degradation was assessed by constructing Arrhenius plots (Fig. 1) to determine the agreement of the experimental data with the Arrhenius model and activation energies. A value for the activation energy for metaldehyde degradation was determined in the pasture clay loam soil over a range of temperatures from 4 to 20 °C. At temperatures <12 °C, the kinetic profile was biphasic, with an initial short period (<3 days) of faster degradation, followed by a slower secondary phase, the latter having a greater influence on the overall rate of dissipation. The Arrhenius plot (Fig. 1) that was constructed with the data fitted with Single First Order (SFO) kinetics demonstrated a greater temperature dependency (E_a_ = 37.5 kJ mol^−1^) than the plot fitted with the second phase of the Double First Order Parallel (DFOP) kinetics (E_a_ = 15.4 kJ mol^−1^). However, the ln of the rate did not depend linearly on 1/T. There was a relatively narrow linear range in each curve, for which the E_a_ was less than the standard value. While the data did not support the hypothesis that the standard temperature correction results in overestimated degradation rates, the data did raise questions about the applicability of Arrhenius theory to metaldehyde degradation in soil. Both derived values suggested a lower temperature dependency for metaldehyde degradation in soil than the standard value (E_a_ = 65.4 kJ mol^−1^) that is used in regulatory risk assessment. These results therefore did not support the initial hypothesis that temperature dependency was a source of error leading to overestimation of degradation rates in soil. This might reflect existing adaptation of the microbial communities to the lower temperature range more typical in their original soil environment (Brown et al., 2002).

**Fig. 2:**
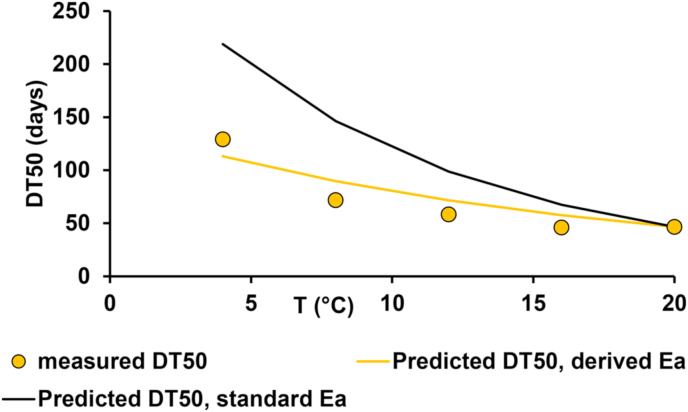
Comparison of measured metaldehyde degradation half-lives (DT_50_ values) in pasture clay loam soil to the metaldehyde degradation half-lives at different temperatures predicted from the rate measured at 20 °C using both the standard activation energy (E_a_) and the experimentally derived activation energy.

The predictions deviated from the experimentally determined values by an appreciable amount for standard activation energy used in regulatory modelling, but not the experimentally derived activation energy using SFO kinetics (Fig. 2). The SFO-derived activation energy was chosen for further modelling because this kinetic model generally fitted the data best. The deviations in the data set in the Arrhenius plots likely represent a change in kinetics towards a more biphasic pattern at lower temperature, which was observed in the degradation profiles of the incubation experiments. All the predicted DT_50_ values in Fig. 2 were calculated from the experimental rate constant measured at 20 °C to replicate the approach used in standard modelling work. Investigations into the suitability of the Arrhenius equation were then expanded to the arable clay loam soil at two moisture contents.

### 3.3 Effects of soil moisture content on metaldehyde degradation

**Fig. 3:**
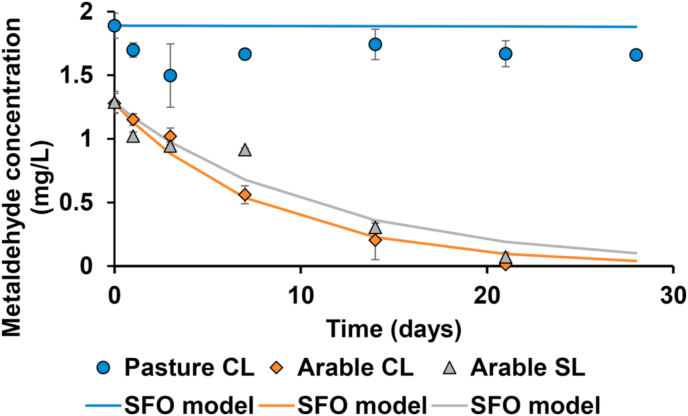
Comparison of metaldehyde degradation profiles in three soils at high soil moisture content of 100% MWHC showing the mean ± 1 standard deviation. The lines show the prediction of the SFO kinetics model.

Of the parameters investigated, soil moisture content had the greatest effect on metaldehyde degradation rate. The degradation profiles for metaldehyde in three soils adjusted to 100% of their maximum water holding capacity are shown in Fig. 3. The most prominent result across the entire suite of batch degradation experiments was that of the pasture clay loam soil under high moisture (100% maximum water holding capacity (MWHC)) conditions where metaldehyde became persistent, with a half-life of 4150 days, far exceeding measured degradation times for any other experiment. On the arable clay loam, the difference was less marked with a DT_50_ of 5.6 d under high soil moisture conditions (100% MWHC) relative to the reference experiment (DT_50_ = 3.0 d; 60% MWHC). Additionally, rapid metaldehyde degradation was observed during experiments using the sandy loam soil, which was also collected field fresh under arable management. Metaldehyde degradation at high soil moisture content (100% MWHC) resulted in faster degradation (DT_50_ = 7.7 d) compared to the reference experiment (10.8 d).

**Fig. 4:**
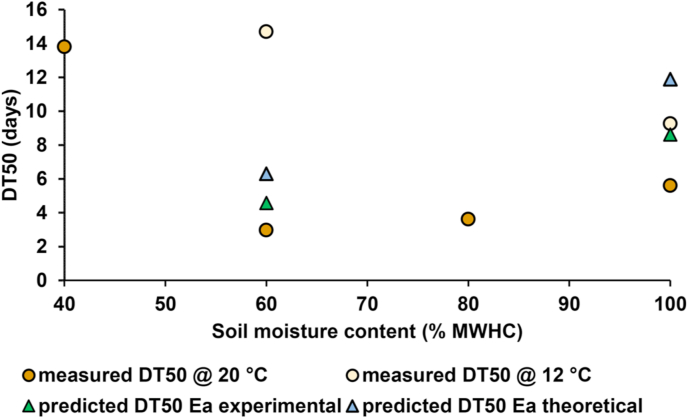
Distribution of metaldehyde biodegradation half-lives (DT_50_) in an arable clay loam soil at different soil moisture contents along with predicted values for 12 °C using the experimentally derived activation energy E_a_ for metaldehyde, and the standard activation energy E_a_.

The combined effect of high soil moisture conditions and lower temperature on degradation rate was measured. Fig. 4 displays how DT_50_s in the arable clay loam soil varied under these two parameters. The data show that DT_50_ was greater when measured at 12 °C, as opposed to 20 °C in soils at two moistures (60 and 100% MWHC), as expected from the temperature-dependency study. However, the higher moisture content soil did not always display slower degradation, as suggested by the hypothesis, and the highest DT_50_ was measured for the driest condition at 40% MWHC.

To examine the predictability of these results, the Arrhenius equation was used to calculate rate constants at 12 °C for metaldehyde degradation occurring at two soil moisture contents: 60% MWHC and 100% MWHC in the arable clay loam soil (Fig. 4). The results generally deviated from the experimental results by an appreciable amount for both the experimentally derived E_a_ and the standard E_a_. The experimentally derived activation energy in this investigation was not substantially more accurate than the standard value, with only the high soil moisture experiment fitting the model well. It was therefore concluded that the activation energy derived for the permanent pasture soil was not applicable to the arable soil of the same texture class, and that derived activation energies were specific to each soil used in this investigation. However, performing multiple temperature-dependency studies in different soils like in this study is an onerous task. Therefore, it was concluded that the standard value for activation energy is a pragmatic approximation for environmental fate modelling in general, because it was in most cases conservative, and better than not accounting for temperature dependency at all.

### 3.4 Metaldehyde degradation at high concentration

**Fig. 5:**
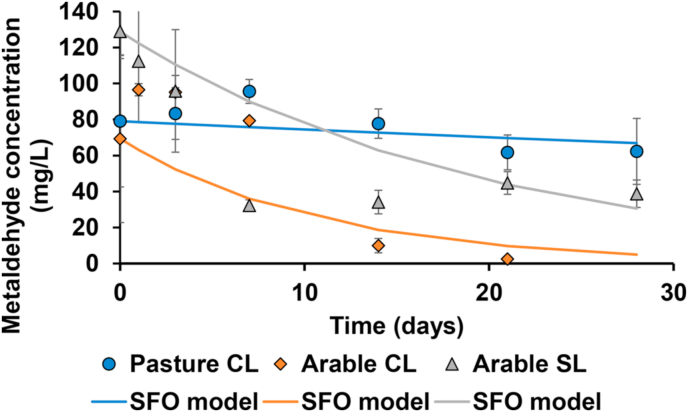
Comparison of metaldehyde degradation profiles in three soils at high metaldehyde soil concentration showing the mean ± 1 standard deviation. The lines show the fit of the SFO kinetics model.

The rate of metaldehyde degradation was highly variable across some batch experiments examining varied metaldehyde soil concentration, where the soil moisture content was kept constant, set to 60% MWHC and an incubation temperature of 20 °C. With reference to Fig. 5, the most outlying result was that of the pasture clay loam conducted with high metaldehyde concentration. The degradation half-life deviated the most from the rest of the data set, with a DT_50_ of 116 days, compared to the reference value of 46.5 days. In all three soils, degradation was slower at the higher spike concentration with a DT_50_ of 7.4 days vs 3 days in the clay loam soil, and a DT_50_ of 13.5 days vs 10.8 days in the sandy loam soil. During these incubations, high metaldehyde concentration resulted in reduced degradation rate.

It was hypothesised that metaldehyde degradation rate would not be affected by high metaldehyde concentration, and the process would continue to follow SFO kinetics. While SFO kinetics was found to be a suitable model, metaldehyde showed a reduced rate of degradation compared to the reference experiment in all three soils. This could be because the microorganisms present in the soil have a finite capacity to degrade metaldehyde when applied to the soil in high concentration, representative of the amount of the active ingredient present in a single metaldehyde pellet spread over a square centimetre of soil. When a growth substrate such as metaldehyde is abundantly available, additional constraints may limit the biodegradation such as nutrient and electron acceptor availability or maximum microbial assimilation and growth rates (Bushnaf et al., 2017).

As in previous experiments, degradation of metaldehyde at high metaldehyde concentration proved faster in the arable clay loam and sandy loam (DT_50_ of 7.4 d and 13.5 d, respectively) than it did in the pasture clay loam. One explanation could be that the abundance of microorganisms capable of degradation metaldehyde is the limiting factor. The microbial community in the arable soils may be conditioned to previous exposure to xenobiotics, hence will degrade synthetic chemical more readily (Yale et al., 2017). Additionally, there are implications of soil type and management, where aeration from cultivation and treatment with fertiliser might enhance the abundance of microorganisms with the capacity to degrade metaldehyde (Hemkemeyer et al., 2015). This might also have a relevance to the observed trends in metaldehyde degradation. The formulation of metaldehyde-based molluscicide as pellets could result in heterogeneous concentrations in the field; metaldehyde might be present in localised pockets of high concentration, where growth controlling factors such as nutrient availability become relevant. In scenarios where metaldehyde concentrations are in gross excess of normal nutrient substrates, following the application of slug pellets, the capacity for the soil microbes to degrade high concentrations of metaldehyde could be limited by their abundance (Ghafoor et al., 2011). If only a fraction of the metaldehyde present in the soil is subjected to the normal SFO degradation processes, this could have implications for the quantities of the chemical leaching to lower soil horizons.

Across the suite of batch incubation experiments, the greatest variability in metaldehyde degradation was generally observed between different soil types, rather than in response to the tested parameter. In the case of the permanent pasture soil, for example, the microbial community of this soil is likely to be conditioned for more anaerobic conditions, created as a result of livestock activity compacting the soil to create a pan and this soil is not disturbed during cultivation; a process which aerates the soil and therefore may facilitate the degradation of metaldehyde, which has been shown to be an aerobic process (EFSA, 2010). The seasonal dynamics of soil microbial communities, as they become habituated to certain environmental conditions might also have an impact on pesticide degradation (Paganin et al., 2010). Analysis of the microbial dynamics associated with metaldehyde degradation represents future work in this project. Microbiology is fundamental to understanding the process of biodegradation, therefore, to improve environmental fate assessments, degradation rates should be measured empirically for appropriate soil types/moisture/temperature conditions in a country, rather than theoretically inferred from data measured under unrepresentative standard conditions.

Metaldehyde exemplifies growing concerns about mobile, persistent, and toxic compounds in the environment and the findings support calls for more robust methods to determine biodegradation rates. This study has illustrated how the compound can become persistent in soil under certain conditions, while sorption studies conducted by regulatory frameworks (EFSA, 2010) demonstrate that in the absence of biodegradation, metaldehyde is only weakly retained in the soil, and can be washed out into surface water. The abundant presence of metaldehyde in surface water confirms this scenario and causes difficulties for the water companies in maintaining stringent drinking water quality standards (Drinking Water Inspectorate, 2018). This study is thus a step towards understanding the reasoning for metaldehyde becoming such a prominent source of pollution in UK surface waters. In addition, there are concerns about the toxicity of metaldehyde pellets to wildlife which have motivated recent UK government decisions on banning the use of metaldehyde (DEFRA, 2020).

## 4 Conclusions

The impact of temperature, moisture content and initial concentration on the degradation rate of metaldehyde in soil varied with soil type. However, a high soil moisture content and high metaldehyde concentration resulted in longer DT_50_ values for metaldehyde than those derived during regulatory assessment for some soils. Based on the results of this study, lack of prior metaldehyde exposure, high moisture content, low temperature and high metaldehyde concentration were identified as high-risk conditions for low pesticide biodegradation in UK soils. It can be easily envisioned how such conditions might locally co-exist in the UK during the metaldehyde application season, and then lead to metaldehyde peaks in surface water affecting drinking water supplies. The findings will have implications for environmental fate modelling and risk assessment, indicating that metaldehyde has the potential to be more persistent in the environment than regulatory assessments might suggest.

## Credit author statement

Nathan Keighley: Methodology, Investigation, Formal analysis, Data curation, Writing – original draft, Visualization. Carmel Ramwell: Conceptualization, Methodology, Validation, Supervision, Writing – review & editing, Funding acquisition. Chris Sinclair: Conceptualization, Methodology, Validation, Supervision David Werner: Conceptualization, Methodology, Formal analysis, Validation, Supervision, Writing – review & editing, Funding acquisition.

## Declaration of competing interest

The authors declare that they have no known competing financial interests or personal relationships that could have appeared to influence the work reported in this paper.

## References

[bib2] Asfaw A., Maher K., Shucksmith J.D. (2018). Modelling of metaldehyde concentrations in surface waters: a travel time based approach. J. Hydrol..

[bib3] Beulke M., Matthies S. (2017). Considerations of temperature in the context of the persistence classification in the EU. Environ. Sci. Europe.

[bib4] Bond S.G. (2018).

[bib5] Brown D.M., Camenzuli L., Redman A.D., Hughes C., Wang N., Viaopoulou E., Saunders D., Villalobos A., Linington S. (2002). Is the Arrhenius correction of biodegradation rates, as recommended through REACH guidance, fit for environmentally relevant conditions? An example from petroleum biodegradation in environmental systems. Sci. Total Environ..

[bib33] Bushnaf K.M., Mangse G., Meynet P., Davenport R.J., Cirpka O.A., Werner D. (2017). Mechanisms of distinct activated carbon and biochar amendment effects on petroleum vapour biofiltration in soil. Environ. Sci. Process. Impacts.

[bib7] Busquets R., Kozynchenko O.P., Whitby R.L.D., Tennison S.R., Cundy A.B. (2014). Phenolic carbon tailored for the removal of polar organic contaminants from water: a solution to the metaldehyde problem?. Water Res..

[bib8] Calumpang S.M.F., Medina M.J., Tejada A.W., Medina J.R. (1995). Environmental impact of two molluscicides: niclosamide and metaldehyde in a rice paddy ecosystem. Bull. Environ. Contam. Toxicol..

[bib9] Castle G.D., Mills G.A., Gravell A., Jones L., Townsend I., Cameron D.G., Fones G.R. (2017). Review of the molluscicide metaldehyde in the environment. Environ. Sci. Water Res. Technol..

[bib10] Castle G.D., Mills G.A., Bakir A., Gravell A., Schumacher M., Snow K., Fones G.R. (2018). Measuring metaldehyde in surface waters in the UK using two monitoring approaches. Environ. Sci. Process. Impacts.

[bib35] DEFRA (2020). https://www.gov.uk/government/news/outdoor-use-of-metaldehyde-to-be-banned-to-protect-wildlife.

[bib14] Dong B., Shao X., Lin H., Hu J. (2017). Dissipation, residues and risk assessment of metaldehyde and niclosamide ethanolamine in pakchoi after field application. Food Chem..

[bib30] Drinking Water Inspectorate (2018).

[bib16] EFSA (2007).

[bib17] EFSA (2010). Conclusion on the peer review of the pesticide risk assessment of the active substance metaldehyde. EFSA J..

[bib1] Ghafoor A., Moeys J., Stenstrom J., Grant T., Jarvis N.J. (2011). Modeling spatial variation in microbial degradation of pesticides in soil. Environ. Sci. Technol..

[bib19] Hale S.E., Arp H.P., Schliebner I., Neumann M. (2020). Persistent, mobile and toxic (PMT) and very persistent and very mobile (vPvM) substances pose an equivalent level of concern to persistent, bioaccumulative and toxic (PBT) and very persistent and very bioaccumulative (vPvB) substances under REACH. Environ. Sci. Eur.

[bib20] Hale S.E., Arp H.P., Schliebner I., Neumann M. (2020). What’s in a name: persistent, mobile and toxic (PMT) and very persistent and very mobile (vPvM). Environ. Sci. Technol..

[bib24] Hemkemeyer M., Christensen B.T., Martens R., Tebbe C.C. (2015). Soil particle size fractions harbour distinct microbial communities and differ in potential for microbial mineralisation of organic pollutants. Soil Biol. Biochem..

[bib26] Kay P, Grayson R (2014). Using water industry data to assess the metaldehyde pollution problem. Water Environ. J..

[bib31] Lu Q., Whitehead P.G., Bussi M.N., Futter M.N., Nizzetto L. (2017). Modelling metaldehyde in catchments: a River Thames case-study. Environ. Sci. Process. Impacts.

[bib23] Met office (2020). https://www.metoffice.gov.uk/research/climate/maps-and-data/summaries/index.

[bib25] Paganin P., Sampedro Pellicer M., Ledda L., Bagella S., Madrau S., Papeleo M.C., Fani R., Dalmastri C., Bevivino A.M. (2010). Soil microbial community response to differences in soil managements and seasonal change. J. Biotechnol..

[bib21] Rüdel H., Körner W., Letzel T., Neumann M., Nödler K., Reemtsma T. (2020). Persistent, mobile and toxic substances in the environment: a spotlight on current research and regulatory activities. Environ. Sci. Europe.

[bib27] Salvestrini S., Vanore P., Bogush A., Mayadevi S., Campos L.C. (2017). Sorption of metaldehyde using granular activated carbon. J. Water Reuse Desalination.

[bib32] Thomas J.C. (2016).

[bib28] Yale R.L., Sapp M., Sinclair C.J. (2017). Microbial changes linked to the accelerated degradation of the herbicide atrazine in a range of temperate soils. Environ. Sci. Pollut. Res..

[bib29] Zhang H.Y., Wang C., Xu P.J., Ma Y.Q. (2011). Analysis of molluscicide metaldehyde in vegetables by dispersive solid-phase extraction and liquid chromatography-tandem mass spectrometry. Food Addit. Contam. A.

